# Diabetic Ocular Surface and Anterior Segment Pathology

**DOI:** 10.1155/2019/5951398

**Published:** 2019-08-14

**Authors:** Valentin Huerva, Francisco J. Ascaso, Andrzej Grzybowski

**Affiliations:** ^1^Department of Ophthalmology, Universitary Hospital Arnau de Vilanova, University of Lleida, Lleida Institute for Biomedical Research Dr. Pifarré Foundation, Avda Rovira Roure 80, 25198 Lleida, Spain; ^2^Department of Ophthalmology, Hospital Clínico Universitario “Lozano Blesa”, Zaragoza, Spain; ^3^Aragon Health Research Institute (IIS Aragon), Zaragoza, Spain; ^4^Department of Ophthalmology, University of Warmia and Mazury, Olsztyn, Poland; ^5^Institute for Research in Ophthalmology, Foundation for Ophthalmology Development, Poznan, Poland

Ocular diabetes has a great morbidity in the world. Diabetic retinopathy (DR) is a well-known vascular entity, and it may be the most frequent cause of visual loss in these patients. Besides the retinal involvement, diabetes mellitus (DM) may affect the anterior ocular segment, especially the ocular surface. Altered corneal sensory nerves and neurotrophic defects are often found in these patients. Likewise, an associated dry eye can also be present. Cataracts are common in the diabetic population. The risk of diabetic macular edema following phacoemulsification surgery is increased. Intravitreal anti-VEGF agents and corticosteroids are used frequently. Nevertheless, these repetitive therapies could be toxic for the corneal endothelium.

Good glycemic control is essential to avoid the appearance of pathological alterations in the previously mentioned territories. The ocular surface serves to help correct diagnosis and follow-up of the diabetic patients. The measurement of glucose concentration in the tear film is a noninvasive test which can show blood glucose changes throughout the day without needing punctures or blood tests.

Confocal microscopy helps to understand what happens in corneal sensory receptors. Different methods to determine the glycemic levels in the tear film have been investigated. 24*-*hour contact lens sensor monitoring is really novel and can be useful in the future. On the other hand, corneal sensitivity may be altered in diabetes due to the systemic polyneuropathy present in DM. The alterations of corneal sensitivity can cause a dry eye due to an absence or reduction of the afferent reflex, and tear osmolarity may be increased.

X. Zeng et al. demonstrated that all lacrimal units may be altered during the course of type 2 DM. Parameters such as SPEED score, Schirmer I test, meibography, Ni-BUT, and lipid layer thickness, among others, worsened with the duration of the disease. G. Qin et al. reported observations about Buddleja officinalis Maxim eye drops in an experimental dry eye rabbit model. These eye drops can improve the morphological structure of the lacrimal gland in castrate dry eye rabbits.

Another highlight of the special issue was reported by Y. Xiao et al. about possible variations between type 1 DM duration and biometric parameters in Chinese children. They found no correlation between HbA1c and duration of the type 1 diabetes and the biometric parameters in children.

Structural and biomechanical corneal differences between diabetics type 2 and nondiabetics patients are reported by J. N. Beato et al. No differences were found in corneal hysteresis and corneal resistance factor between both groups.

K. Krysik et al. reported a pachymetric evaluation in diabetic patients with Scheimpflug camera and swept-source optical coherence tomography. They compared pachymetry in diabetics type 2 and nondiabetics individuals.

An update on corneal biomechanics and architecture in DM is reviewed by M. A. del Buey et al. The epithelium, stroma, endothelium, and corneal nerves suffer specific complications during the disease.

Finally, a study by D. S. Lomoriello et al. shows that an early subclinical alteration in subbasal nerve corneal plexus is detected by confocal microscopy in absence of other diabetic complications, including microvascular diabetic complications, diabetic peripheral neuropathy, diabetic autonomic neuropathy, diabetic retinopathy, and microalbuminuria.

In summary, this special issue offers an overview of the alterations of DM in the ocular surface and the anterior ocular segment. It also provides new findings that may be clue for new research in this important field.

